# Carbon dioxide and hydrogen as building blocks for a sustainable interface of energy and chemistry

**DOI:** 10.1098/rsta.2023.0266

**Published:** 2024-09-23

**Authors:** Walter Leitner

**Affiliations:** ^1^ Max Planck Institute for Chemical Energy Conversion, Mülheim an der Ruhr, Germany; ^2^ Institute for Technical and Macromolecular Chemistry, RWTH Aachen University, Aachen, Germany

**Keywords:** Green Chemistry, sustainable energy

## Abstract

Hydrogen as energy vector from renewable sources and carbon dioxide as carbon source are central elements of a future sustainable interface between energy and chemistry. While often viewed merely as “substitutes” for fossil resources, the current article discusses opportunities to open new synthetic pathways and to generate novel molecular architectures for the delivery of the same or even improved functionalities expected from chemical products. Catalysis is the key science and technology in this endeavour and three general principles for the desing of catalytic systems are proposed as guidelines for fundamental research.

This article is part of the discussion meeting issue ‘Green carbon for the chemical industry of the future’.

## 1. The vision

**Figure 1.**
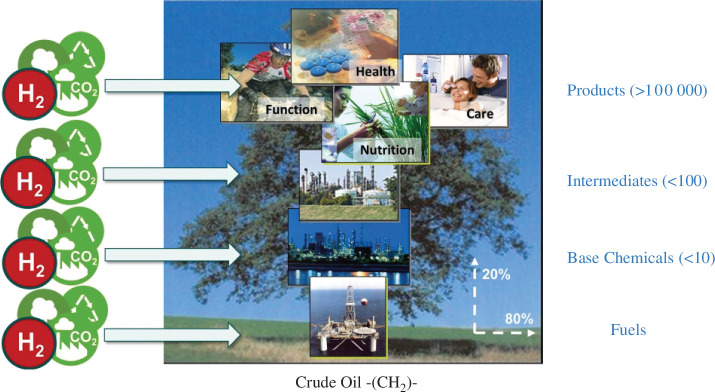
Translation of the fossil-based linear value chain into a carbon cycle by (re-)introducing renewable biomass, recycled materials and CO_2_ at various stages of the ‘ChemisTree’. Today, *ca* 100 Mio barrels (1 barrel = 160 l) per day of crude oil provide the main feedstock for the production of fuels and base chemicals, with a ratio of approximately 80% fuels and 20% chemicals. This ratio is expected to shift towards the need for chemicals owing to (i) direct electrification in the mobility sector and (ii) increased demand for chemical products to ensure prosperity for a growing human population.

Crude oil *fuels* the Anthropocene—literally—through the production of liquid energy carriers for mobility and transportation as well as by providing the crucial source of carbon as the essential element for the chemical value chain. Despite worldwide efforts to reduce greenhouse gas emissions, the demand for crude oil is predicted to reach an all-time high in the coming years and forecasts for reduction require a range of measures centred around the global availability of renewable energy. The resulting *de-carbonization* of the energy sector imposes challenges but also holds many opportunities for the *de-fossilization* of the sectors’ mobility and chemistry, where direct electrification is difficult or even impossible. Ensuring access to nutrition, health care, education and equality for a growing population in a sustainable manner will continue to require carbon-based energy carriers, products and services. Translating the concept of a *fossil carbon-based value chain* to a *renewable carbon cycle* requires a drastic paradigm shift. Rather than mitigating CO_2_ as undesired waste once the carbon has fulfilled its function, it can be re-integrated as feedstock through catalytic processes combining it with hydrogen obtained from decarbonized technologies (figure 1) [1–5].

## 2. The mission

Today, worldwide activities in academia and industry are already directed towards the replacement of petroleum-based fuels and chemicals with substitutes produced from biomass, recycled material or carbon dioxide. For industrial purposes, it seems attractive to achieve the transition while minimizing possible changes to existing process networks to maximize the retention of existing assets and supply chains [6]. However, the fundamental change of the energy input and carbon source offers the potential to align the transition with the principles of green chemistry to address sustainability criteria beyond greenhouse gas emission [7,8]. Thus, the challenge to generate low-carbon fuels and chemicals simultaneously holds the opportunity to aim for better processes and products. In other words, a major driver for change can be innovation.

**Figure 2.**
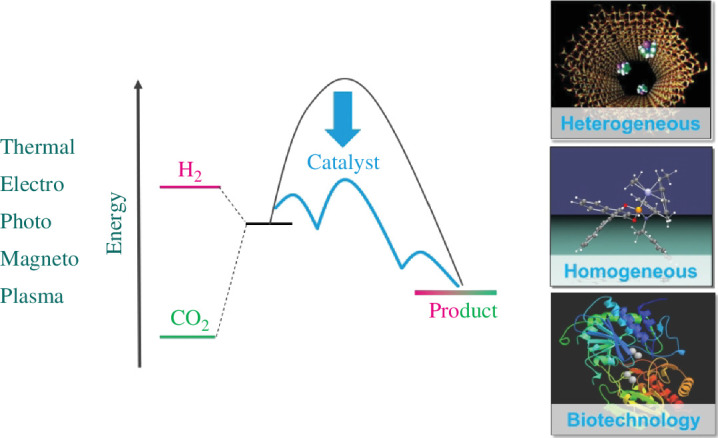
The catalytic disciplines provide the essential toolbox for the utilization of CO_2_ and H_2_, and alternative forms of energy input can be envisaged beyond thermal catalysis. The thermodynamic stability of CO_2_ requires an additional high-energy partner to ensure a sufficient thermodynamic driving force, and the catalyst opens mechanistic pathways to overcome the otherwise insurmountable kinetic barriers.

The central science and technology used to exploit the full potential of this historical chance is catalysis (figure 2) [9]. The traditional disciplines of catalysis should not be viewed as competitors for individual transformations, but rather as a toolbox to achieve optimum solutions in a systems approach. On a fundamental level, the common principles and their distinct manifestations for the activation of hydrogen, CO_2_ and potential co-substrates are yet to be fully deciphered. The integration with energy supply beyond thermal catalysis can influence the overall efficacy substantially if the scalability and robustness of actual production schemes can be assured.

## 3. The objectives

The scientific interest in using CO_2_ as a carbon source has a long tradition. A major driver was, and still is, the fundamental interest in nature’s manifold to capture and convert carbon dioxide through photosynthesis. The role of CO_2_-based equilibria was studied owing to its technical importance in major industrial processes using CO for the production of energy carriers and base chemicals with heterogeneous catalysts. The discovery that CO_2_ can bind as a ligand in stable organometallic complexes stirred a dynamic area of research in homogeneous catalysis which started in the late 1970s. It was in particular the organometallic catalysis community that recognized the potential of CO_2_ as a building block beyond the formation of C1 products such as methanol and methane [10]. As early as 1991, the Max Planck Society funded a working group ‘CO_2_ Chemistry’ at the University of Jena in Germany. Initially in the field, transformations of CO_2_ with energy-rich molecules such as epoxides, alkynes or olefins were in the focus, as hydrogen was considered to be available only from steam reforming of methane with concomitant generation of CO_2_—clearly defeating the purpose of the concept. The dynamic deployment of cost-effective technologies for power generation from renewable sources has been a game changer in this context.

**Figure 3.**
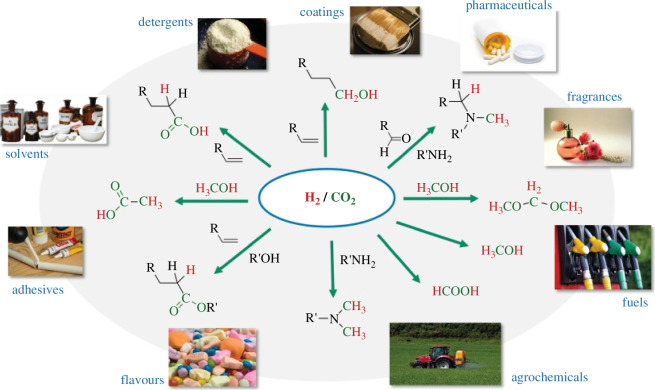
Connecting the desired function of products with molecular architectures and synthetic pathways for their generation using CO_2_ as a carbon source (green) and hydrogen as reduction equivalents (red).

Major scientific progress in organometallic catalysis has been made during the last 10–15 years, recognizing the potential of CO_2_/H_2_ as a building block for the synthesis not only of C1 products, but also for base chemicals, commodities and even complex structures as required in fine chemicals, consumer care and even pharmaceutical products [11]. Figure 3 highlights some of these novel transformations, illustrating in the products the carbon from CO_2_ in green and the hydrogens from H_2_ in red [12]. The figure also exemplifies the possibility to connect the desired function of a product with certain molecular architectures that can then be traced back to the renewable building blocks of carbon dioxide and hydrogen. Utilizing this concept of ‘retrosynthetic analysis’, well known in traditional organic synthesis, has previously been proposed for biomass utilization and can equally be applied to CO_2_/H_2_. In fact, the ultimate goal in achieving closed carbon cycles is to also sample the co-substrates from biomass or recycled materials to ensure that no additional fossil carbon would be employed in the synthetic pathways. While primarily mono-functionalized products are exemplified in figure 3, the same principle can be applied to the synthesis of monomers for the production of important classes of polymers such as polyesters, polyamides, polycarbonates or polyurethanes [13].

To turn the challenges of the transition from fossil to renewable into strategic opportunities for innovation, we have identified three main objectives for the research in our team. With each objective, there is a leading reference provided for further reading.

### (a) From cracking to building

Whereas the key entry points into the petrochemical value chain are processes to crack C–C and C–H bonds in the long chain energy-rich molecules, synthetic pathways starting from CO_2_ rely on the combination with H_2_ to build up molecular complexity from small molecules [14]. Thus, the molecular design of individual chemical products and even multi-component mixtures, as in fuels, comes into reach.

### (b) From analytic to predictive

The synthetic transformations using organometallic catalysis in the petrochemical value chain are well established and there is a deep understanding of the underlying catalytic cycles from experiment and theory in many cases [15]. Can this analytical knowledge of the molecular mechanisms be translated into predictive approaches to use computational chemistry, potentially aided by algorithm-based methods, for the identification of catalysts or lead structures for currently *unknown* transformations?

### (c) From optimized to adaptive

Specific processes of the petrochemical value chain are typically based on catalysts that have been optimized for a particular task to operate largely under static conditions that maximise the output of a single product. With the coupling to renewable, and hence fluctuating, energy sources and increasing diversity and variation of feedstock qualities, catalytic systems that can be adjusted or even that self-adjust adaptively to dynamic changes in real-time may become attractive alternatives [16]. This includes the possibility of flexible customized production of different products from a single starting material reacting, for example, on variations in energy availability or costs.

## 4. The perspective

The goal of net-zero or even carbon-negative production of chemicals and molecular energy carriers can only be achieved if the carbon is not released as waste that needs to be mitigated, but we strive for the vision of a closed anthropogenic carbon cycle. In this context, the use of CO_2_ and H_2_ as building blocks for new synthetic pathways is one of the key components [17]. It is not the ‘silver bullet’, but complements as essential technology other strategies such as biomass utilization or plastic recycling. Notably, the combination of such feedstocks can elevate on a systems level the limitations of the individual approaches such as large energy demand, land use or distributed availability.

On a fundamental level, progress in catalysis science and process engineering is urgently required to not only assist the transition towards renewables but also to shape the future of the energy-chemistry nexus to be truly sustainable. We hope that the objectives outlined in the technical section of this perspective can serve as general guidelines in this area. The incredibly dynamic change from the field of catalytic CO_2_ conversion being viewed as an almost esoteric curiosity in the late twentieth century to one of the most active fields of catalysis research in the early twenty-first century certainly lends hope to the aspiration that the ingenuity and innovation of chemists will contribute to this development. In the words of Nelson Mandela, *It always seems impossible—until it’s done*.

## Data Availability

This article has no additional data.

## References

[1] Aresta M . 2010 Carbon dioxide as chemical feedstock. Weinheim, Germany: Wiley-VCH.

[2] Centi G , Perathoner S . 2009 Opportunities and prospects in the chemical recycling of carbon dioxide to fuels. Catal. Today **148** , 191–205. (10.1016/j.cattod.2009.07.075)

[3] Sakakura T , Choi JC , Yasuda H . 2007 Transformation of carbon dioxide. Chem. Rev. **107** , 2365–2387. (10.1021/cr068357u) 17564481

[4] Artz J , Müller TE , Thenert K , Kleinekorte J , Meys R , Sternberg A , Bardow A , Leitner W . 2018 Sustainable conversion of carbon dioxide: an integrated review of catalysis and life cycle assessment. Chem. Rev. **118** , 434–504. (10.1021/acs.chemrev.7b00435) 29220170

[5] Group of Chief Scientific Advisors, Directorate-General for Research and Innovation . 2018 Novel carbon capture and utilisation technologies. Brussels, Belgium: European Commission. (10.2777/01532)

[6] DECHEMA and Furure CAMP on behalf of VCI . 2020 Working towards a climate neutral chemical industry in Germany. See https://www.vci.de/startseite.jsp.

[7] Poliakoff M , Leitner W , Streng ES . 2015 The twelve principles of CO2 CHEMISTRY. Faraday Discuss. **183** , 9–17. (10.1039/c5fd90078f) 26528836

[8] Zimmerman JB , Anastas PT , Erythropel HC , Leitner W . 2020 Designing for a green chemistry future. Science **367** , 397–400. (10.1126/science.aay3060) 31974246

[9] Klankermayer J , Leitner W . 2016 harnessing renewable energy with CO_2_for the chemical value chain: challenges and opportunities for catalysis. Phil. Trans. R. Soc. A **374** , 20150315. (10.1098/rsta.2015.0315) 26755762

[10] Behr A . 1987 Use of carbon dioxide in industrial organic syntheses. Chem. Eng. Technol. **10** , 16–27. (10.1002/ceat.270100103)

[11] Klankermayer J , Wesselbaum S , Beydoun K , Leitner W . 2016 Selective catalytic synthesis using the combination of carbon dioxide and hydrogen: catalytic chess at the interface of energy and chemistry. Angew. Chem. Int. Ed. **55** , 7296–7343. (10.1002/anie.201507458) 27237963

[12] Klankermayer J , Leitner W . 2015 Love at second sight for CO_2_ and H_2_ in organic synthesis. Science **350** , 629–630. (10.1126/science.aac7997) 26542554

[13] Langanke J , Wolf A , Hofmann J , Böhm K , Subhani MA , Müller TE , Leitner W , Gürtler C . 2014 Carbon dioxide (CO_2_) as sustainable feedstock for polyurethane production. Green Chem. **16** , 1865–1870. (10.1039/C3GC41788C)

[14] Voelker S *et al* . 2024 Towards carbon-neutral and clean propulsion in heavy-duty transportation with hydroformylated fischer–tropsch fuels. Nat. Energy. (10.1038/s41560-024-01581-z)

[15] Kemper G , Hölscher M , Leitner W . 2023 Pd(II)-catalyzed carboxylation of aromatic C-H bonds with CO_2_ . Sci. Adv. **9** , eadf2966. (10.1126/sciadv.adf2966) 36735781 PMC9897662

[16] Bordet A , Leitner W . 2023 Adaptive catalytic systems for chemical energy conversion. Angew. Chem. Int. Ed. Engl. **62** , e202301956. (10.1002/anie.202301956) 37345624

[17] Leitner W , Schmitz M . 2021 Concluding remarks: carbon dioxide utilization: where are we now? and where are we going? Faraday Discuss. **230** , 413–426. (10.1039/d1fd00038a) 34223853

